# Association between IL-1β and recurrence after the first epileptic seizure in ischemic stroke patients

**DOI:** 10.1038/s41598-020-70560-7

**Published:** 2020-08-11

**Authors:** Qingyan Zhang, Guanghong Li, Duanyun Zhao, Peng Yang, Tuerxun Shabier, Tuerhong Tuerxun

**Affiliations:** 1 Department of Neurointensive Care Unit, The First Affiliated Hospital of Xinjiang Medical University, No. 137, Liyushan Nan Road, Ürümqi, 830054 China; 2 Department of Neurosurgery, Heze Municipal Hospital of Shandong Province, No. 2888, Caozhou Road, Mudan District, Heze, 274000 China; 3 Department of Neurosurgery, The People’s Hospital of Lanling County, Huibao Road, Lanling County, 277799 China

**Keywords:** Biomarkers, Risk factors

## Abstract

To analyze the association of IL-1β with recurrence after the first epileptic seizure in ischemic stroke patients and evaluate its predictive value. 238 patients with the first epileptic seizure after ischemic stroke were included in this study. IL-1β expression levels were detected through quantitative Real-Time PCR. Kaplan–Meier method was used to perform univariate analysis with log-rank test. The variables with *P* < 0.1 were then included in multivariate analysis. Receiver operating characteristic (ROC) curve was used to evaluate the predictive value. Among all 238 patients, 107 patients (44.96%) had seizure recurrence and 131 patients (55.04%) had no recurrence. Kaplan–Meier analysis showed that high expression of IL-1β, low age (< 65 years), male, cortical involvement, large lesion size, late onset, severe neurological impairment and partial seizure type were associated with seizure recurrence. Multivariate analysis showed that IL-1β expression level (hazard ratio 2.057, 95% confidence interval 1.296–3.318) was independently associated with seizure recurrence. The area under ROC curve (AUC) was 0.803 (SE 0.030, 95% confidence interval 0.744–0.862) when IL-1β expression levels were applied in predicting seizure recurrence. IL-1β might be a useful biomarker for early discovery of recurrence after the first epileptic seizure in ischemic stroke patients.

## Introduction

Epilepsy is a multifaceted neurologic disorder characterized with recurrent seizures which are induced by paroxysmal uncontrolled discharges of neurons^1–3^. The majority of persons with epilepsy are from the developing countries^4,5^. A recent report shows that the lifetime prevalence of active epilepsy is 7.60 per 1,000 persons while the point prevalence of active epilepsy was 6.38 per 1,000 persons^6^. Stroke is a common cause of epilepsy, particularly in elderly population. Almost 50% of epileptic seizures happen for the first time as a result of ischemic stroke in patients with an age greater than 60 years^7^. The incidence of post-stroke seizures varies widely, ranging from 2 to 20%^8–10^. Despite of advances in anti-epileptic drugs, about 30% of epilepsy patients are still refractory to medical treatment, demonstrating progressive cognitive impairment, and may need neurosurgical resection of the epileptic focus to reduce seizure recurrence^11^. Post-stroke seizures are associated with additional complications, increased mortality and longer initial hospitalization, as well as affecting quality of life of patients during rehabilitation after stroke^12,13^. Therefore, the timely discovery and treatment of epileptic seizures is critical to promote rehabilitation of stroke patients.

IL-1β is a constitutively expressed inflammatory cytokine in the central nervous system (CNS)^14^. It has neurotrophic factor-like activity^15^ or modulates both the activity and expression of ion channels in the CNS^16^. IL-1β is significantly upregulated after ischemic stroke^17–21^, and elevated IL-1β level has been reported in multiple forms of epilepsy with different etiologies^22^. Moreover, experimental models have demonstrated that intracerebral application of IL-1β may enhance electrographic seizures^23^. However, to date, the association between IL-1β level and recurrence after the first epileptic seizure has not been analyzed in ischemic stroke patients, moreover, the value of IL-1β level applied in predicting seizure recurrence has not been evaluated. In this study, we analyzed the association between IL-1β level and recurrence after the first epileptic seizure in ischemic stroke patients using Kaplan–Meier analysis and Cox regression model, and evaluated the value of IL-1β level in predicting seizure recurrence with ROC curves. The aim was to provide a useful biomarker for early discovery of recurrence after the first epileptic seizure in ischemic stroke patients.

## Results

### General data

There were 139 males and 99 females among the 238 patients with the first epileptic seizure after ischemic stroke. Their median age was 65 years with interquartile range of 54–73 years at the first epileptic seizure. Their median follow-up duration was 37 months with interquartile range of 23–60 months after ischemic stroke and 23 months with interquartile range of 13–39 months after the first epileptic seizure. Among them, 107 patients (44.96%) had seizure recurrence and 125 patients (55.04%) had no recurrence. Detailed characteristics were shown in Table 1.

**Table 1:** General data of 238 patients with the first epileptic seizure after ischemic stroke.

	No	Percentages (%)
**Gender**		
Male	139	58.40
Female	99	41.60
Smoking	128	53.78
Drinking	28	11.76
Hypertension	153	64.29
Diabetes mellitus	100	42.02
Atrial fibrillation	60	25.21
Coronary heart disease	98	41.18
Cortical involvement	85	35.71
Hemorrhagic transformation	57	23.95
**Lesion size**		
Small	47	19.75
Moderate	69	28.99
Large	122	51.26
**EEG findings**		
Normal	121	50.84
Abnormal	117	49.16
**Seizure type**		
Generalized	123	51.68
Partial	115	48.32
**Time of seizure**		
Early onset	114	47.90
Late onset	124	52.10
**Neurological impairment**		
Mild and moderate	140	58.82
Severe	98	41.18
Status epilepticus	21	8.82
Seizure recurrence	107	44.96

### IL-1β expression level

The IL-1β expression level of all 238 patients was 4.72 (2.05–7.13). Mann–Whitney U test demonstrated that the IL-1β expression level was higher in patients with seizure recurrence than in patients without recurrence (6.49 vs 3.18, n = 238, *P* < 0.05).

### Univariate analysis

**Figure 1:**
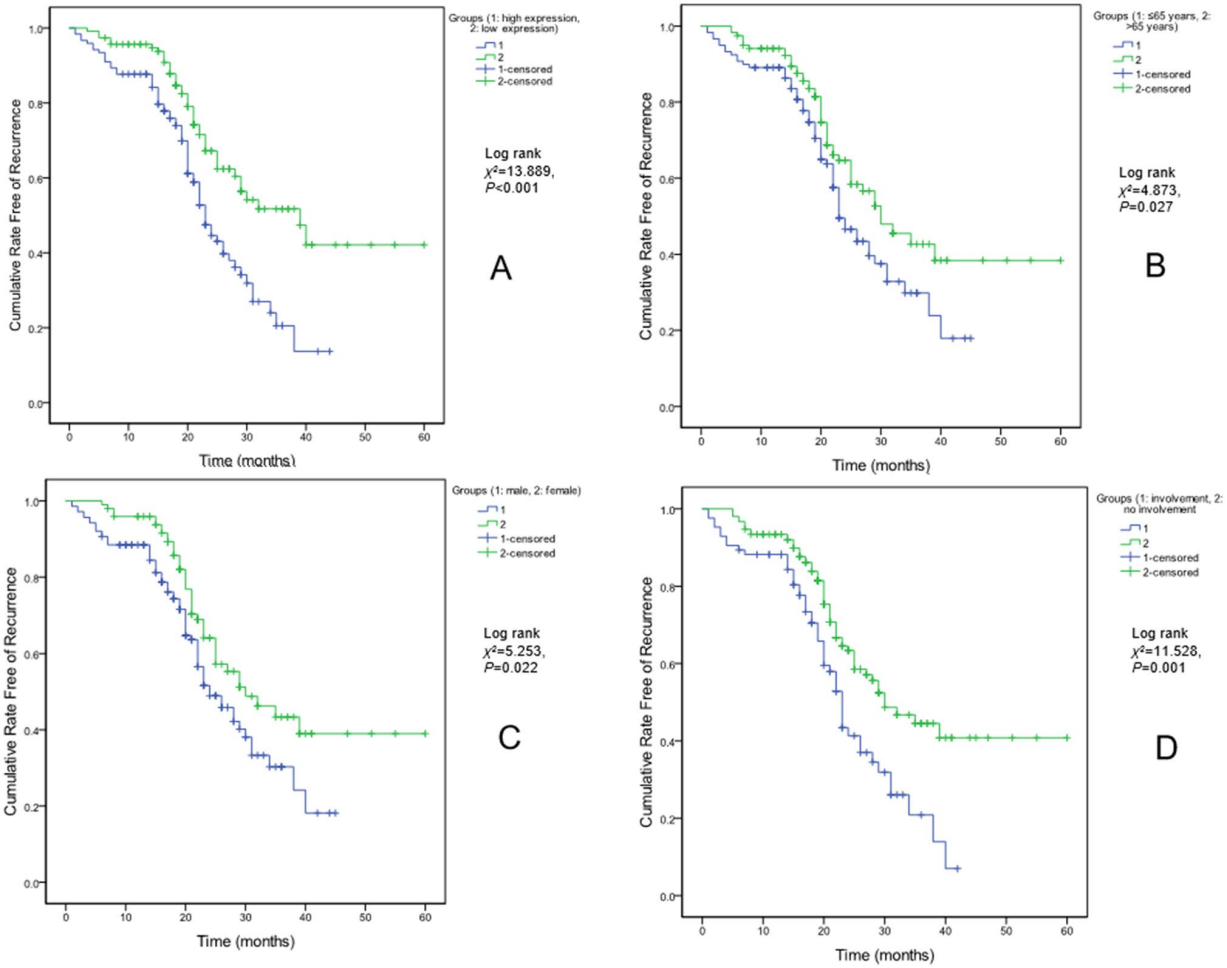
Kaplan–Meier analysis for cumulative rate free of seizure recurrence. (**a**) for IL-1β expression level, (**b**) for age, (**c**) for gender, and (**d**) for cortical involvement.

**Figure 2:**
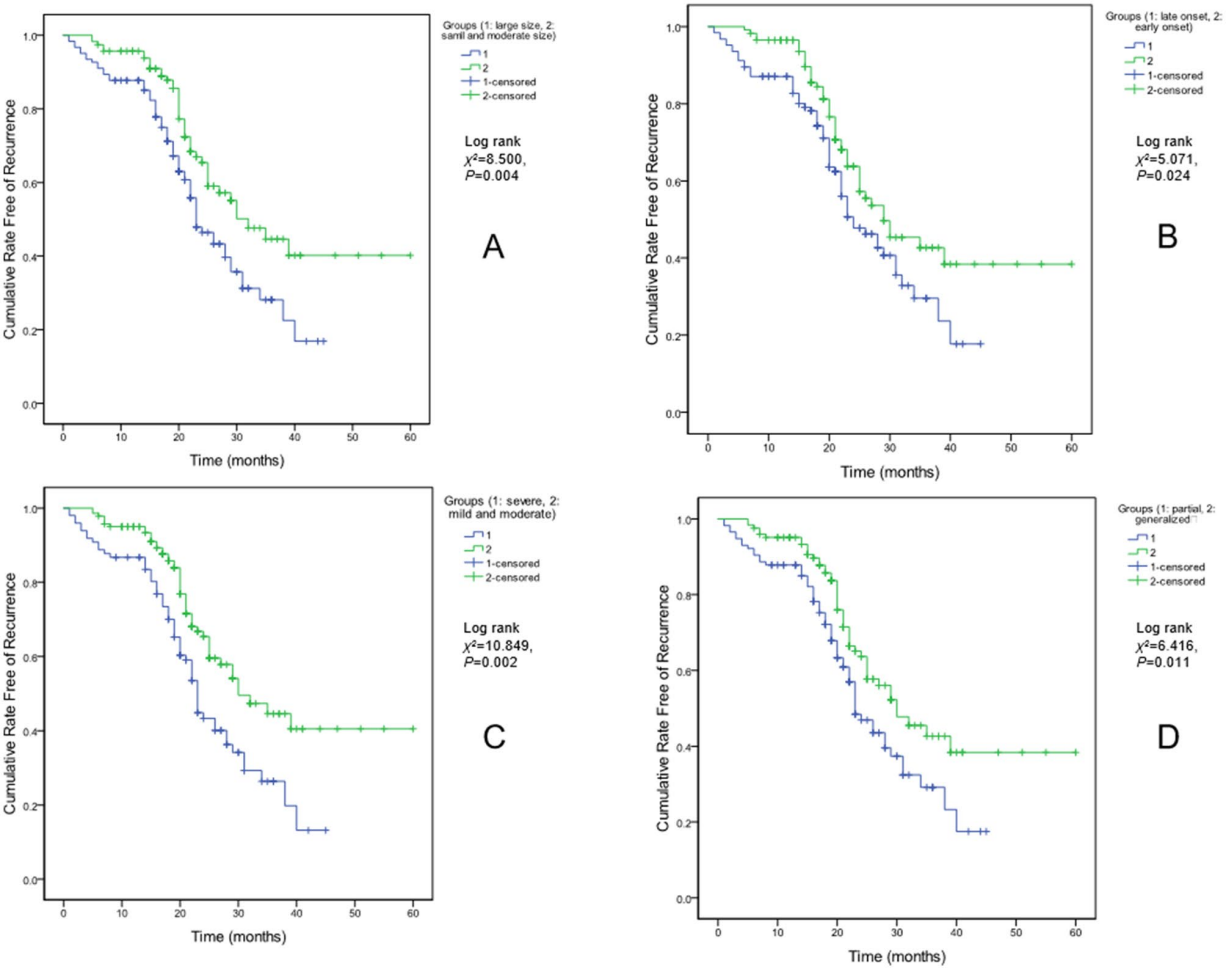
Kaplan–Meier analysis for cumulative rate free of seizure recurrence. (**a**) for lesion size, (**b**) for onset time, (**c**) for neurological impairment, and (**d**) for seizure type.

The 238 patients were grouped into low and high expression groups based on the median expression level of IL-1β. According to Kaplan–Meier analysis, high expression of IL-1β was associated with seizure recurrence (Fig. 1a). In addition, Kaplan–Meier analysis demonstrated that low age (< 65 years) (Fig. 1b), male (Fig. 1c), cortical involvement (Fig. 1d), large lesion size (Fig. 2a), late onset (Fig. 2b), severe neurological impairment (Fig. 2c) and partial seizure (Fig. 2d) were associated with seizure recurrence.

### Multivariate analysis

The IL-1β expression level, age, gender, cortical involvement, lesion size, time of seizure, neurological impairment, seizure type, hemorrhagic transformation and EEG findings were included in Cox regression model. Multivariate analysis showed that the IL-1β expression level was independently associated with seizure recurrence after adjusting for age, gender, cortical involvement, time of seizure, neurological impairment, seizure type, hemorrhagic transformation and EEG findings (Table 2). The *hazard ratio* of IL-1β expression level was 2.057 (95% *confidence interval*: 1.296–3.318), greater than 1. Therefore, IL-1β was an independent risk factor for recurrence of epileptic seizure, i.e., the elevated IL-1β expression level could increase the risk of recurrence after the first epileptic seizure in ischemic stroke patients.

**Table 2:** Results of multivariate analysis with Cox regression model.

	Regression coefficient	Standard error	Wald *χ*^2^	Hazard ratio	95% confidence interval	*P*
IL-1β	0.874	0.312	6.386	2.057	1.296–3.318	0.009
Low age	0.618	0.293	4.898	1.839	1.205–3.124	0.030
Male	0.917	0.439	5.229	1.995	1.271–3.147	0.022
Cortical involvement	1.113	0.458	6.015	2.402	1.414–3.922	0.012
Large lesion size	0.962	0.357	5.473	2.196	1.327–3.586	0.017
Late onset	0.776	0.315	5.086	1.872	1.219–3.049	0.025
Severe neurological impairment	1.028	0.436	5.857	2.371	1.388–3.853	0.014
Partial seizure	0.915	0.371	5.392	2.073	1.293–3.394	0.019
Hemorrhagic transformation	0.489	0.226	2.785	1.644	0.878–2.896	0.127
Abnormal EEG findings	0.517	0.241	2.926	1.703	0.895–2.917	0.118

### Predictive value of IL-1β expression levels

**Figure 3:**
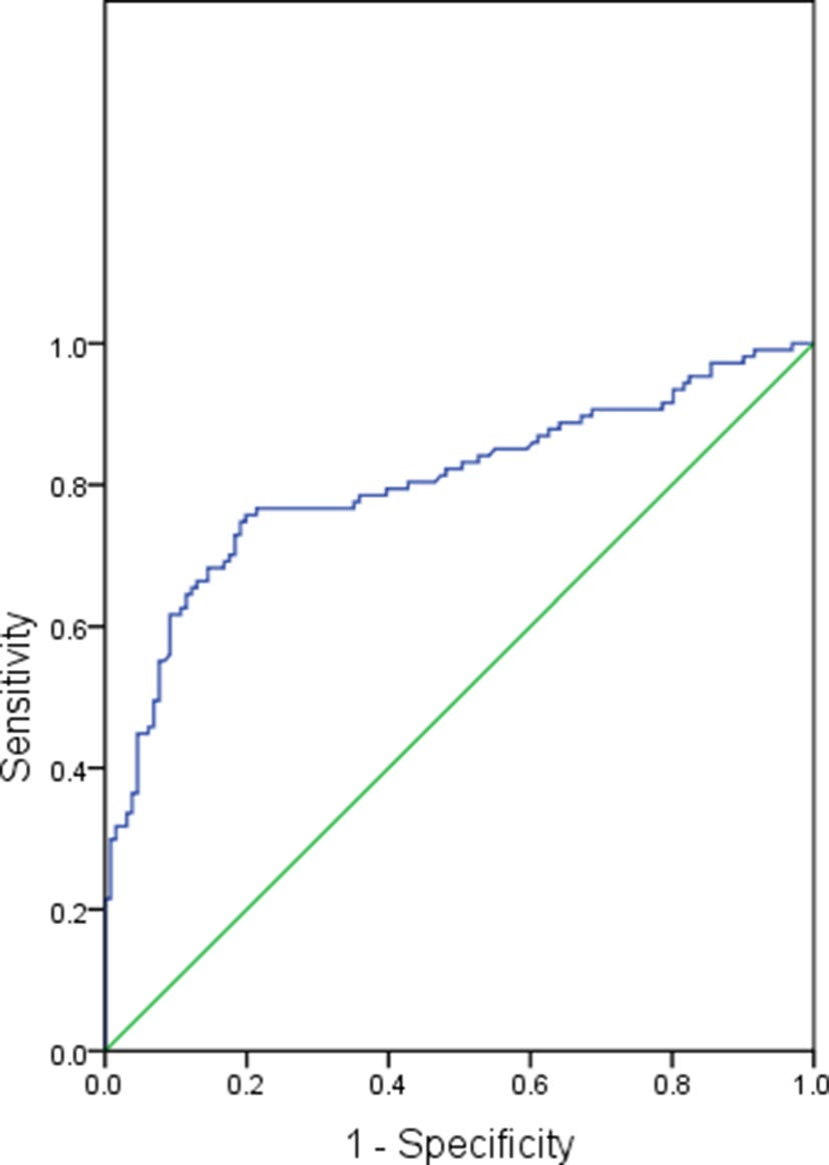
ROC curve of IL-1β expression levels applied in predicting recurrence after the first epileptic seizure in ischemic stroke patients.

The area under ROC curve (AUC) was 0.803 (SE 0.030, 95% confidence interval 0.744–0.862) when IL-1β expression levels were applied in predicting seizure recurrence (Fig. 3). Therefore, the predictive value was high. The optimal cutoff value was 5.42. When the optimal cutoff value was used as the prediction criterion, i.e., recurrence was determined when > 5.42 and no recurrence when ≤ 5.42. The predictive results were demonstrated in Table 3. The sensitivity, specificity, accuracy, false positive rate, false negative rate, positive predictive value and negative predictive value were 70.09%, 87.02%, 79.41%, 18.49%, 21.92%, 81.52% and 78.08%, respectively.

**Table 3:** Predictive results of IL-1β expression levels at the optimal cutoff value.

	Recurrence	No recurrence	Total
Positive (> 5.42)	75	17	92
Negative (≤ 5.42)	32	114	146
Total	107	131	238

## Discussion

Inflammation is a critical contributor to brain injury caused by stroke, and inflammatory mediators are associated with prognosis of stroke patients^24–27^. Post-stroke inflammation is generally regulated by microglia and astrocytes in the brain^28,29^. Under the protection of the blood–brain barrier, microglia plays a role in maintaining healthy brain function via producing growth/repair factors, pruning synapses and clearing debris^30^. Microglia are activated after stroke, obviously transforming their morphology to a large, amoeboid structure from a thin, ramified state^31^, which is demonstrated to be accompanied by production of inflammatory mediators. Plenty of clinical and experimental studies show that neuroinflammation characterized with upregulated expression of inflammatory mediators via activated microglia is^32^ associated with the pathological process of epilepsy^33–38^. Neuroinflammation is a shared characteristic in epileptic foci of the human brain and experimental models^33–36^. Moreover, the conditions which cause neuroinflammation and production of inflammatory mediators can facilitate epileptogenesis^37,38^.

IL-1β, a pro-inflammatory cytokine produced primarily by activated microglia and astrocytes, is one of important inflammatory mediators during neuroinflammation after stroke. As a constitutively expressed inflammatory cytokine in the CNS^14^, IL-1β is typically in low levels in the brain and elevates significantly via activation of microglia and astrocytes after stroke^17–21^. Elevated IL-1β levels have been reported in multiple forms of epilepsy with different etiologies, suggesting its role in initiation and progression of epilepsy^22^.

In clinical studies, Ichiyama et al. found that IL-1β levels were significantly upregulated in cerebrospinal fluid of patients with febrile seizures^39^; Vezzani et al. indicated that the secretion and production of IL-1β were upregulated in serum and cerebrospinal fluid of patients with epilepsy after tonic–clonic seizures^40^; Uludag et al.^41^ demonstrated that serum IL-1Ra, IL-1β and IL-6 levels were significantly increased in patients with temporal lobe epilepsy or extra-temporal lobe epilepsy, and moreover serum IL-1β level was also significantly elevated in patients with temporal lobe epilepsy; Shi et al. also showed that IL-1β levels were significantly elevated in cerebrospinal fluid in the epileptic pediatric population compared with the controls^42^; Roseti et al. indicated that upregulated levels of IL-1β in temporal lobe epilepsy could decrease GABA-mediated neurotransmission and lead to generation of seizures due to neuronal hyper-excitability^43^.

In experimental models, upregulated expression of IL-1β was also detected in epileptogenic tissues from animals with epilepsy of different etiologies^44,45^. Studies showed that mRNA expression of IL-1β, VEGF, TNF-α and TGF-β1 was significantly upregulated in the hippocampus after seizures^46–48^. Ho et al. demonstrated that peripheral inflammation induced by LPS could increase seizure susceptibility via upregulation of IL-1β, TNF-α and IL-6^49^; Auvin et al. found that IL-1β receptor antagonist could partially reverses the enhancement of epileptogenesis in immature rat brains^44^; Xiao et al.^50^ reported that IL-1β level was associated with the epileptogenesis of mesial temporal lobe epilepsy, and the mechanism was that IL-1β might induce activation of mammalian target of rapamycin (mTOR), followed by activation of neurons, which was crucial for the pathogenesis of mesial temporal lobe epilepsy chronicity; Viviani et al. found that IL-1β could upregulate NMDA receptors on postsynaptic cells through activating the GluN2B subunit of the NMDA receptor, which was associated with induction of seizures^16^; Postnikova et al. showed that expression of the GluN2B mRNA upregulated significantly at 24 h following seizures, which might result in impairment of synaptic plasticity^51^. In addition, IL-1β also contributes to breakdown of the blood–brain barrier, while breakdown of the blood–brain barrier has been shown to be an indispensable component of epileptogenesis following brain injury^52^. For all these reasons, we propose such a hypothesis that IL-1β is associated with recurrence after the first epileptic seizure in ischemic stroke patients. Our results showed that the IL-1β expression level was higher in patients with seizure recurrence than in patients without recurrence; and multivariate analysis further showed that the IL-1β expression level was independently associated with seizure recurrence after adjusting for potential confounders, i.e., the elevated IL-1β expression level could increase the risk of recurrence after the first epileptic seizure in ischemic stroke patients. These results validated our hypothesis. Additionally, we also evaluated the value of IL-1β in predicting seizure recurrence, and the results showed that its predictive value was high with an AUC of 0.803. The sensitivity, specificity, accuracy, false positive rate, false negative rate, positive predictive value and negative predictive value were 70.09%, 87.02%, 79.41%, 18.49%, 21.92%, 81.52% and 78.08%, respectively.

Therefore, IL-1β might be a useful biomarker for early discovery of recurrence after the first epileptic seizure in ischemic stroke patients.

## Materials and methods

### Patients

A total of 3,296 consecutive patients with ischemic stroke were retrospectively collected in The First Affiliated Hospital of Xinjiang Medical University and Heze Municipal Hospital between June 2013 and June 2018. The first epileptic seizure after ischemic stroke occurred in 259 patients (7.86%), including 122 patients with early onset and 137 patients with late onset. All 259 patients were followed up for 1 ~ 5 years, and 8 patients died and 13 patients were lost of follow up. Finally, 238 patients with the first epileptic seizure after ischemic stroke were included in this study. Among them, 107 patients (44.96%) had seizure recurrence and 131 patients (55.04%) had no recurrence. This study was permitted by the ethic committee of First Affiliated Hospital of Xinjiang Medical University (201,503,061,228), and all the experiment protocol for involving humans was in accordance to guidelines of national/international/institutional or Declaration of Helsinki. Written informed consents were provided by patients or legally authorized representatives.

### Inclusion and exclusion criteria

Inclusion criteria included (1) patients with first-ever ischemic stroke that was determined through symptoms and signs, computed tomography (CT) or magnetic resonance imaging (MRI), and medical histories; (2) patients with the first epileptic seizure after ischemic stroke that was diagnosed according to the criteria suggested by Berg et al. and Fisher et al.^2,53^; and (3) patients with complete medical histories.

Exclusion criteria included (1) patients with primary hemorrhagic stroke or transient ischemic attack; (2) patients with a history of epilepsy or family history of epilepsy; (3) patients with probable epileptogenic lesions such as traumatic brain injury, brain tumor, brain surgery, cerebral hemorrhage, cerebral vascular malformation and cortical dysplasia; (4) non-convulsive electroencephalographic (EEG) seizure; and (5) patients who died within 2 weeks after ischemic stroke.

### Data collection

All patients were investigated for demographic data (gender and age), risk factors of cerebrovascular disease (smoking, drinking, hypertension, diabetes mellitus, atrial fibrillation and coronary heart disease), CT or MRI imaging data (cortical involvement, hemorrhagic transformation and lesion size), EEG findings, NIHSS scores, time and type of the first epileptic seizure, and whether status epilepticus or not.

### Definitions

Seizure recurrence was defined as the second unprovoked seizure that was apart from the first one by more than 24 h through the definition of epilepsy^2,53^. Generalized seizures were defined as originating at some point within, and rapidly engaging, bilaterally distributed networks; and partial seizures were defined as originating within networks limited to one hemisphere. EEG findings were divided into normal and abnormal, and abnormal findings included generalized slow, regional slow and epileptiform discharge. Lesion size was divided into large (> 50 × 50 mm and > 5 slices), moderate (≤ 15 × 15 mm and > 5 slices or > 50 × 50 mm and ≤ 5 slices) and small (≤ 15 × 15 mm and ≤ 5 slices). Neurological impairment was assessed using National Institutes of Health Stroke Scale (NIHSS) scores (< 16 for mild and moderate, and ≥ 16 for severe). Early onset was defined as the first epileptic seizure occurring within 2 weeks after ischemic stroke and late onset as the first epileptic seizure occurring outside 2 weeks.

### Detection of IL-1β expression levels

The miRCURY RNA Isolation Kit—Biofluids was employed to isolate total RNA (Exiqon, Vedbaek, Denmark). The RNA 6,000 Pico Kit (Agilent, Santa Clara, USA) was used to evaluate the quality of the isolated RNA through an Agilent 2,100 Bioanalyzer (Agilent, Santa Clara, USA). The total RNA was reversely transcribed to cDNA with the Reverse Transcriptase Kit (M-MLV) (Zomanbio, Beijing, China). IL-1β expression levels were detected using quantitative Real-Time PCR (qRT-PCR). Rotor-Gene Q Real-Time Fluorescence Quantitative PCR Analyzer (Qiagen, Germany) was employed to conduct qRT-PCR using a Qiagen kit and a TaqMan universal PCR master mix. GADPH was selected as the reference gene, and the 2^−△△Ct^ method was used to evaluate the expression levels of IL-1β. The primers were manually designed with the online NCBI primer-BLAST tool and chemosynthesized by Shanghai Jima Biotech Ltd (Shanghai, China). The primers of IL-1β were 5′-TGATGGCTTATTACAGTGGCAATG-3′ (forward) and 5′-GTAGTGGTGGTCGGAGATTCG-3′ (reverse), and the primers of GADPH were 5′-AGCCTCAAGATCAGCAATG-3′ (forward) and 5′-CACGATACCAAAGTTGTCATGGAT-3′ (reverse).

### Statistical analysis

All statistical analysis was conducted through the SPSS version 17.0 for Windows (SPSS Inc., USA). Categorical data were shown as ratios or percentages (%). The normality of continuous data was validated using Kolmogorov–Smirnov test. Non-normal data were shown as median and interquartile range, and normal data were shown as mean ± standard deviation (SD). Kaplan–Meier method was employed to conduct univariate analysis with log-rank test. The variables with *P* < 0.1 in univariate analysis were then included in multivariate analysis. Cox regression model was employed in multivariate analysis. Receiver operating characteristic (ROC) curve was employed to assess the value of IL-1β expression level in predicting seizure recurrence. Significance was set at two sided *P* < 0.05.

## Data Availability

All data generated or analysed during this study are included in this published article.
